# Supplementary material for: Plasma membrane transbilayer asymmetry of PI(4,5)P2 drives unconventional secretion of Fibroblast Growth Factor 2
Source: Nat Commun. 2025 Nov 29;16:10816. doi: 10.1038/s41467-025-66860-z (PMC12669245; doi:10.1038/s41467-025-66860-z)
Supplement: Supplementary file 1 — Supplementary information [file 41467_2025_66860_MOESM1_ESM.pdf]

Materials

Supplementary table 1. Lipids used for vesicles preparation

Lipid	Order number	Company
Liver PC	840055P	Sigma Avanti
Liver PE	840026P/840026C	Sigma Avanti
Brain PS	840032P/840032C	Sigma Avanti
Liver PI	840042P/840042C	Sigma Avanti
Brain PI(4,5)P2	840046P	Sigma Avanti
Brain PI(4)P	840045P	Sigma Avanti
Egg SM	860061P	Sigma Avanti
Cholestrol	700000P	Sigma Avanti
BiotynlPE	870282P/870282C	Sigma Avanti
16:0 Liss RhPE	810158P	Sigma Avanti
18:0/20:4 PI(3,4)P_2	850188P	Sigma Avanti
18:0/20:4 PI(3,4,5)P_3	850166P	Sigma Avanti

*All the lipid were purchased from avanti and stored under Argon

Supplementary table 2. Reagents/Fluorophores/Proteins and antibodies used in this study

Item	Order number	Company
LC-Heparin	H3149-10KU	Sigma
Sucrose	4621.1	Roth
Biotin-BSA	A8549	Sigma
Neutravidin	A2666	Invitrogen/ThermoFisher
Alexa647	A33084	Invitrogen/ThermoFisher
PIP5K1C	A33522	ThermoFisher
$MgCl_2$	63068	Fluka
$CaCl_2$	31307	Sigma
HEPES	H4034	Sigma
KCl	SZBG3080H	Fluka
KOH	30603	Sigma
ATP	10 519 979 001	Roche
EDTA	1.08418.1000	Sigma
BSA fatty free	REF 10775 835 001	Roche
Paraformaldehyde 16% solution (PFA)	15210	Electron Microscopy Science
Alpha-MEM	M8042	Sigma

Dulbecco PBS	D8537	Sigma
Live cells imaging solution (LCI)	A14291DJ	ThermoFisher
Halo Ligand-Alexa Fluor 660	G847B	Promega
Halo Ligand-Alexa Fluor 488	G1001	Promega
AntiGFP-Alexa647 (rabbit polyclonal)	A-31852 (LOT no. 2836757)	ThermoFisher
PHX2-Halo-Halo	-	
His-FGF2-GFP	-	
His-FGF2-Halo	-	
AnnexinV-Alexa647	A23204	ThermoFisher
