# Supplementary material for: Plasma membrane transbilayer asymmetry of PI(4,5)P2 drives unconventional secretion of Fibroblast Growth Factor 2
Source: Nat Commun. 2025 Nov 29;16:10816. doi: 10.1038/s41467-025-66860-z (PMC12669245; doi:10.1038/s41467-025-66860-z)
Supplement: Supplementary file 2 — Description of Additional Supplementary File [file 41467_2025_66860_MOESM2_ESM.pdf]

Description of Additional Supplementary Files

Supplementary movie 1: PM + 5mol% PI(4,5)P2 GUV treated with Mg2+, ATP, PIP5K1C and tracer dye Alexa-647 for 15mins: Giant unilamellar vesicles (GUVs) were prepared with 5 mol% PI(4,5)P_2 along with membrane marker Rhodamine-PE, in PM like background. After immobilizing the GUVs, Mg2+, ATP, PIP5K1C and small trace dye Alexa-647 was added and time was marked as 0 minute. Vesicle was monitored for the entire course of kinase reaction i.e. 15 minutes. (Scale bar = 10µm)

Supplementary movie 2: PM + 5mol% PI(4)P GUV treated with Mg2+, ATP, PIP5K1C and tracer dye Alexa-647 for 15mins: Giant unilamellar vesicles (GUVs) were prepared with 5 mol% PI(4)P along with membrane marker Rhodamine-PE, in PM like background. After immobilizing the GUVs, Mg2+, ATP, PIP5K1C and small trace dye Alexa-647 was added and time was marked as 0 minute. Vesicle was monitored for the entire course of kinase reaction i.e. 15minutes. (Scale bar = 10µm)

Supplementary movie 3: FGF2-GFP pore formation kinetics for symmetric PM + 5mol% PI(4,5)P2 GUV in real time: Giant unilamellar vesicles (GUVs) were prepared with 5 mol% PI(4,5)P_2 along with membrane marker Rhodamine-PE, in PM like background. Small trace dye Alexa-647 was added to record the event of pore formation for kinetic measurement. After addition of FGF2-GFP, time was marked as 0 minute and vesicle was given 10-20 minutes to immobilize before starting the time series. (Scale bar = 10µm)

Supplementary movie 4: FGF2-GFP pore formation kinetics for asymmetric PI(4,5)P2 GUV in real time: Giant unilamellar vesicles (GUVs) were prepared with 5 mol% PI(4)P along with membrane marker Rhodamine-PE, in PM like background. Vesicles were subjected to kinase reaction with Mg2+ + ATP + PIP5K1C for conversion of PI(4)P to PI(4,5)P_2 to yield asymmetric vesicle. Following the kinase reaction, vesicles were washed to remove unreacted components or damaged vesicles. Small trace dye Alexa647 was added to record the event of pore formation for kinetic measurement. After addition of FGF2-GFP, time was marked as 0 minute and vesicle was given 10-20 minutes to immobilize before starting the time series. (Scale bar = 10µm)

Supplementary movie 5: FGF2-GFP pore formation kinetics for symmetric PM + 5mol% PI(4,5)P2 GUV in real time: Giant unilamellar vesicles (GUVs) were prepared with 5 mol% PI(4,5)P_2 along with membrane marker Rhodamine-PE, in PM like background. Vesicles were treated with Mg2+ + ATP + PIP5K1C as a control. Following the kinase reaction, vesicles were washed to remove unreacted components or damaged vesicles. Small trace dye Alexa-647 was added to record the event of pore formation for kinetic measurement. After addition of FGF2-GFP, time was marked as 0 minute and vesicle was given 10-20 minutes to immobilize before starting the time series. In the presented video, the time series starts at 23 minutes. However, since the vesicle was not immobilized until 33 minutes, the cropped video

excludes the 23–33 minutes segment. Please refer to the uncropped video for the full time series sequence. (Scale bar = 10μm)
