# Supplementary material for: Plasma membrane transbilayer asymmetry of PI(4,5)P2 drives unconventional secretion of Fibroblast Growth Factor 2
Source: Nat Commun. 2025 Nov 29;16:10816. doi: 10.1038/s41467-025-66860-z (PMC12669245; doi:10.1038/s41467-025-66860-z)
Supplement: Supplementary file 8 — Reporting summary [file 41467_2025_66860_MOESM8_ESM.pdf]

nature portfolio

Corresponding author(s): Fabio Lolicato and Walter Nickel

Last updated by author(s): Nov 9, 2025

Reporting Summary

Nature Portfolio wishes to improve the reproducibility of the work that we publish. This form provides structure for consistency and transparency in reporting. For further information on Nature Portfolio policies, see our Editorial Policies and the Editorial Policy Checklist.

Statistics

For all statistical analyses, confirm that the following items are present in the figure legend, table legend, main text, or Methods section.

n/a	Confirmed	
☐	☒	The exact sample size (n) for each experimental group/condition, given as a discrete number and unit of measurement
☐	☒	A statement on whether measurements were taken from distinct samples or whether the same sample was measured repeatedly
☐	☒	The statistical test(s) used AND whether they are one- or two-sided<br>*Only common tests should be described solely by name; describe more complex techniques in the Methods section.*
☒	☐	A description of all covariates tested
☐	☒	A description of any assumptions or corrections, such as tests of normality and adjustment for multiple comparisons
☐	☒	A full description of the statistical parameters including central tendency (e.g. means) or other basic estimates (e.g. regression coefficient) AND variation (e.g. standard deviation) or associated estimates of uncertainty (e.g. confidence intervals)
☐	☒	For null hypothesis testing, the test statistic (e.g. F, t, r) with confidence intervals, effect sizes, degrees of freedom and P value noted<br>*Give P values as exact values whenever suitable.*
☒	☐	For Bayesian analysis, information on the choice of priors and Markov chain Monte Carlo settings
☒	☐	For hierarchical and complex designs, identification of the appropriate level for tests and full reporting of outcomes
☒	☐	Estimates of effect sizes (e.g. Cohen's d, Pearson's r), indicating how they were calculated

Our web collection on statistics for biologists contains articles on many of the points above.

Software and code

Policy information about availability of computer code

Data collection	Zen 2.6 (Zeiss Confocal Microscopy), Image Studio 5.2.5 (LI-COR Gel Scanning), BD CellQuest Pro 6.0 (Flow Cytometry). All the raw data are provided as a source data zip file and codes used, concerning this study are provided as DOI.
Data analysis	For data analysis of confocal images, we use in-house macros script for imageJ and available at DOI: https://doi.org/10.5281/zenodo.17306345. All the graphs were plotted in graphPad Prism along with the statistical analysis. All the raw data are provided as a source data zip file and codes used, concerning this study are provided as DOI.

For manuscripts utilizing custom algorithms or software that are central to the research but not yet described in published literature, software must be made available to editors and reviewers. We strongly encourage code deposition in a community repository (e.g. GitHub). See the Nature Portfolio guidelines for submitting code & software for further information.

Data

Policy information about availability of data

All manuscripts must include a data availability statement. This statement should provide the following information, where applicable:

- Accession codes, unique identifiers, or web links for publicly available datasets
- A description of any restrictions on data availability
- For clinical datasets or third party data, please ensure that the statement adheres to our policy

Data Availability

All source data associated with this study are provided in the accompanying Source Data zip file. Uncropped gel images and original video files are also included. Details of the corresponding data files are specified in each figure legend. The Fiji macros used in this study are publicly available from Zenodo under accession code 10.5281/zenodo.17306345.

Code Availability

The Fiji macros used in this study have been deposited in the Zenodo repository under accession code 10.5281/zenodo.17306345. The Fiji macros are publicly available without restriction.

Research involving human participants, their data, or biological material

Policy information about studies with human participants or human data. See also policy information about sex, gender (identity/presentation), and sexual orientation and race, ethnicity and racism.

Reporting on sex and gender	n/a
Reporting on race, ethnicity, or other socially relevant groupings	n/a
Population characteristics	n/a
Recruitment	n/a
Ethics oversight	n/a

Note that full information on the approval of the study protocol must also be provided in the manuscript.

Field-specific reporting

Please select the one below that is the best fit for your research. If you are not sure, read the appropriate sections before making your selection.

☒ Life sciences ☐ Behavioural & social sciences ☐ Ecological, evolutionary & environmental sciences

For a reference copy of the document with all sections, see nature.com/documents/nr-reporting-summary-flat.pdf

Life sciences study design

All studies must disclose on these points even when the disclosure is negative.

Sample size	Sample size for every reported experiment is provided in manuscript text, methods section and figure legend. Usually, each experiment was done ≥ 3 times independently. For experiment where we need a single cell/GUV analysis to compute bulk population parameters, ≥15 Individual events were recorded. For each replicate 15-100 cell or 19-135 single GUVs were taken to compute general population parameters.
Data exclusions	No data has been excluded
Replication	Every reported experiment has been replicated three or more times and this information is provided in manuscript text, methods section and figure legend
Randomization	n/a
Blinding	As the sample size was small and very diverse with different controls, blinding wasn't feasible or required.

Reporting for specific materials, systems and methods

We require information from authors about some types of materials, experimental systems and methods used in many studies. Here, indicate whether each material, system or method listed is relevant to your study. If you are not sure if a list item applies to your research, read the appropriate section before selecting a response.

Materials & experimental systems

n/a	Involved in the study	
☐	☒	Antibodies
☐	☒	Eukaryotic cell lines
☒	☐	Palaeontology and archaeology
☒	☐	Animals and other organisms
☒	☐	Clinical data
☒	☐	Dual use research of concern
☒	☐	Plants

Methods

n/a	Involved in the study	
☒	☐	ChIP-seq
☐	☒	Flow cytometry
☒	☐	MRI-based neuroimaging

Antibodies

Antibodies used	AntiGFP-Alexa647, rabbit polyclonal, A-31852, (LOT no. 2836757) ThermoFisher used at 1:400 dilution. Information for materials used is also provided in material and methods sections.
Validation	According to the manufacturer: "This Antibody was verified by Relative expression to ensure that the antibody binds to the antigen stated." (section "advanced verification" on the website: https://www.thermofisher.com/antibody/product/GFP-Antibody-Polyclonal/A-31852)

Eukaryotic cell lines

Policy information about cell lines and Sex and Gender in Research

Cell line source(s)	We used CHO-K1-FGF2GFP cells derived from CHO-K1 cells. Parent cell lines, CHO-K1, were received from the DSMZ - Deutsche Sammlung von Mikroorganisem und Zellkulturen GmbH (German collection of microorganisms and cell cultures GmbH), DSMZ no.:ACC 110. DSMZ states that these cells are "subclone from parental CHO cell line that was initiated from an ovary biopsy of an adult Chinese hamster in 1957."
Authentication	CHO-K1-FGF2GFP cells, identity and purity were analyzed by a multiplex cell contamination test. Multiplex cell contamination test was done on 18-March-2022 before using the cell line. (Schmitt and Pawlita, 2009).
Mycoplasma contamination	Cell line tested negative for mycoplasma contamination using multiplex cell contamination test. Additionally, cells were always treated with mycoplasma removal agent for a week before use.
Commonly misidentified lines (See ICLAC register)	CHO-K1 (Chinese hamster ovary) cell line used in this study is not listed among the misidentified cell lines by International Cell Line Authentication Committee (https://iclac.org/databases/cross-contaminations/).

Plants

Seed stocks	n/a
Novel plant genotypes	n/a
Authentication	n/a

Flow Cytometry

Plots

Confirm that:

☒ The axis labels state the marker and fluorochrome used (e.g. CD4-FITC).

☒ The axis scales are clearly visible. Include numbers along axes only for bottom left plot of group (a 'group' is an analysis of identical markers).

☐ All plots are contour plots with outliers or pseudocolor plots.

☐ A numerical value for number of cells or percentage (with statistics) is provided.

Methodology

Sample preparation	FACS was done on large unilamellar vesicles containing rhodamine-PE lipid. Detailed procedure is mentioned in methods section "FACS flow cytometry". FACS procedures were adapted from Temmerman and Nickel, 2009 (https://doi.org/10.1194/jlr.D800043-JLR200)
Instrument	FACSCalibur Becton Dickinson
Software	FACS data were handled as done in Temmerman and Nickel, 2009 (https://doi.org/10.1194/jlr.D800043-JLR200)
Cell population abundance	No cells used, only large unilamellar vesicles were use and FACS procedures were adapted from Temmerman and Nickel, 2009 (https://doi.org/10.1194/jlr.D800043-JLR200)
Gating strategy	Liposomes were gated on the basis of size and fluorescence (https://doi.org/10.1194/jlr.D800043-JLR200)

☐ Tick this box to confirm that a figure exemplifying the gating strategy is provided in the Supplementary Information.
