# Supplementary figures and images for: Plasma membrane transbilayer asymmetry of PI(4,5)P2 drives unconventional secretion of Fibroblast Growth Factor 2
Source: Nat Commun. 2025 Nov 29;16:10816. doi: 10.1038/s41467-025-66860-z (PMC12669245; doi:10.1038/s41467-025-66860-z)

Cropped images

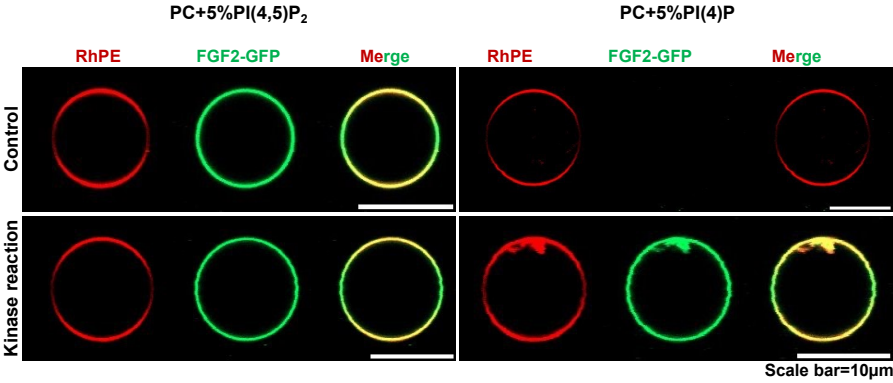

Uncropped images

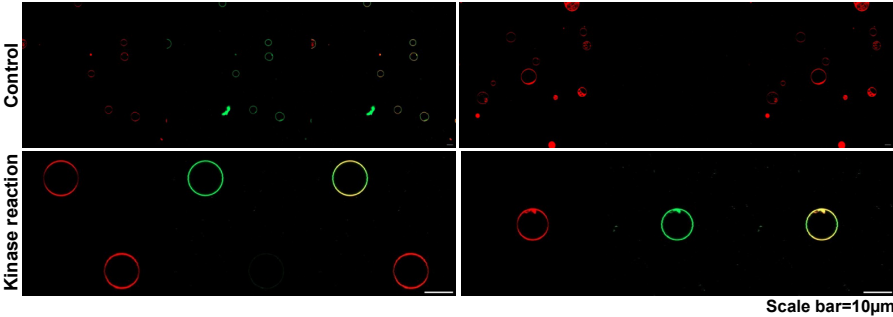

Supplement: Supplementary file 10 — SourceData [file 41467_2025_66860_MOESM10_ESM.zip › SourceData_NCOMMS-25-36735A/SorceData Figure 1 Cropped-Uncropped Confocal Images panel d and e.pdf]

Cropped images

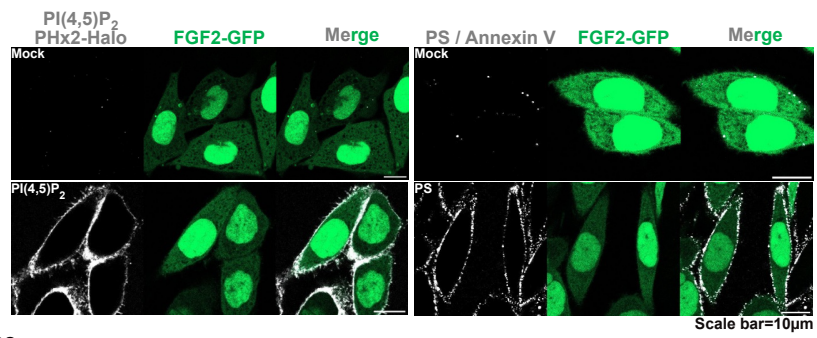

Uncropped images

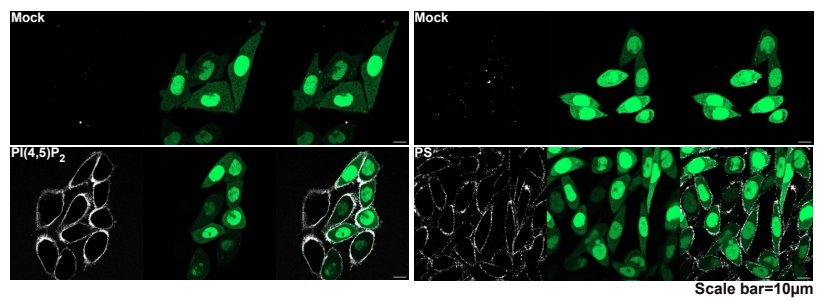

Supplement: Supplementary file 10 — SourceData [file 41467_2025_66860_MOESM10_ESM.zip › SourceData_NCOMMS-25-36735A/SorceData Figure 6 Cropped-Uncropped Confocal Images panel b.pdf]

# Cropped images

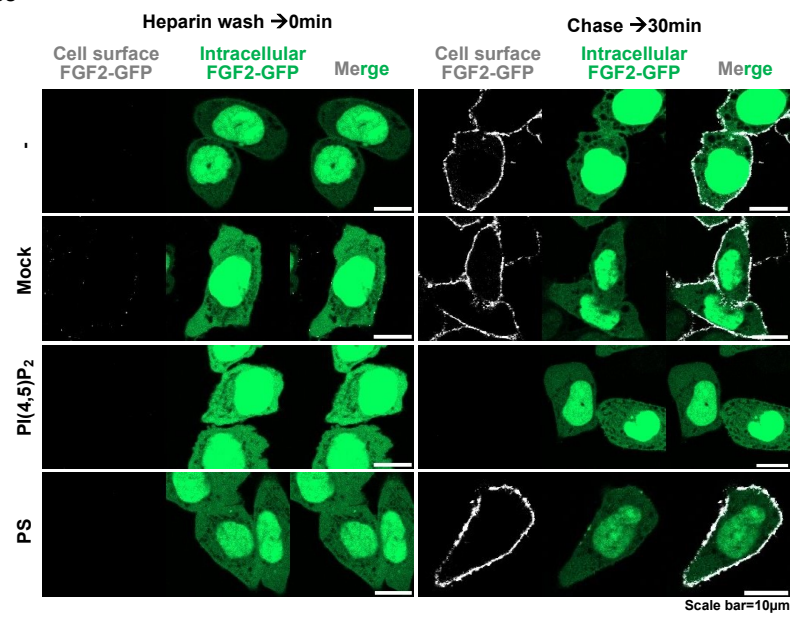

# Uncropped images

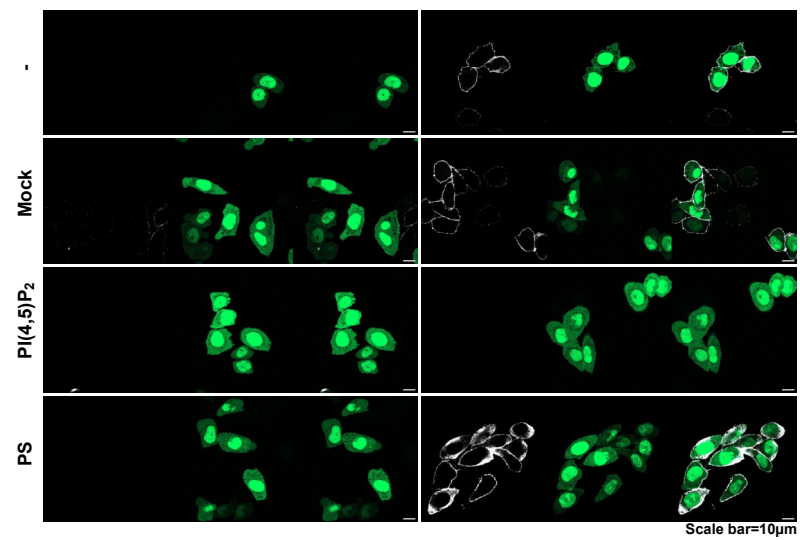

Supplement: Supplementary file 10 — SourceData [file 41467_2025_66860_MOESM10_ESM.zip › SourceData_NCOMMS-25-36735A/SorceData Figure 8 Cropped-Uncropped Confocal Images panel a.pdf]

Cropped images

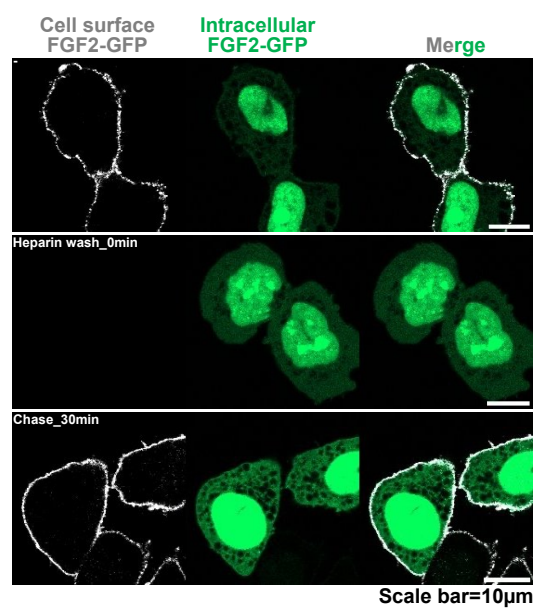

Uncropped images

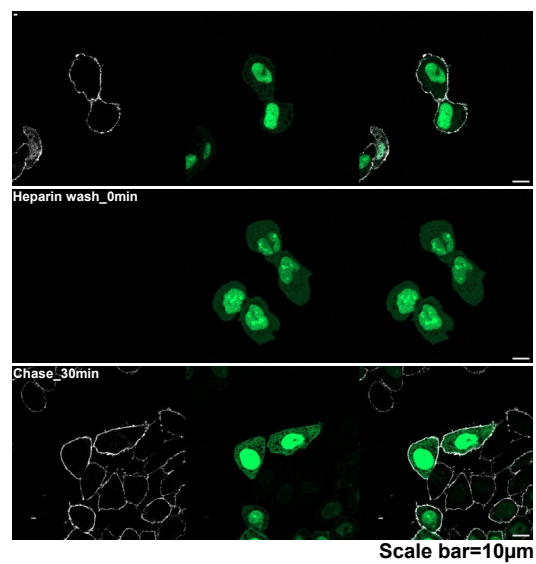

Supplement: Supplementary file 10 — SourceData [file 41467_2025_66860_MOESM10_ESM.zip › SourceData_NCOMMS-25-36735A/SorceData Figure 7 Cropped-Uncropped Confocal Images.pdf]

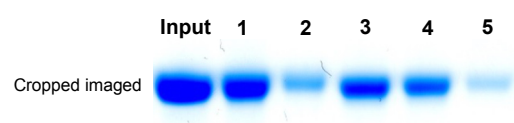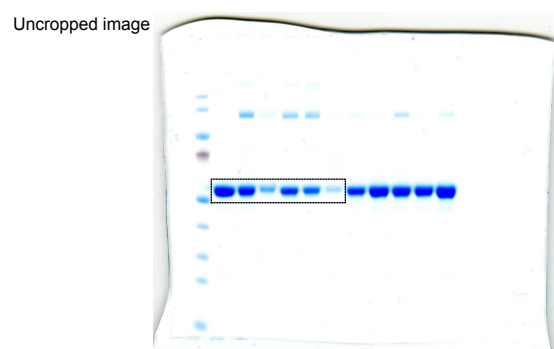

Supplement: Supplementary file 10 — SourceData [file 41467_2025_66860_MOESM10_ESM.zip › SourceData_NCOMMS-25-36735A/SourceData Figure 3 Cropped-Uncropped gel panel a.pdf]

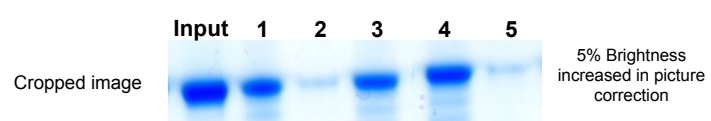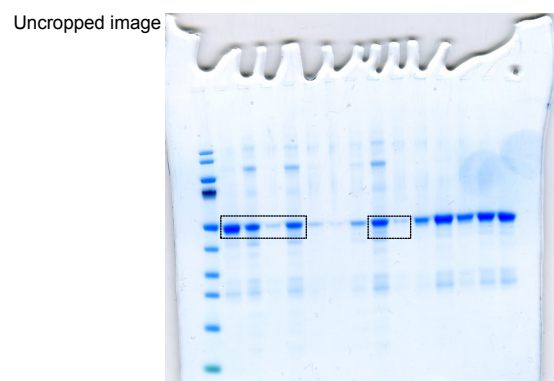

Supplement: Supplementary file 10 — SourceData [file 41467_2025_66860_MOESM10_ESM.zip › SourceData_NCOMMS-25-36735A/SourceData Figure 1 Cropped- Uncropped gel panel c.pdf]

Cropped images

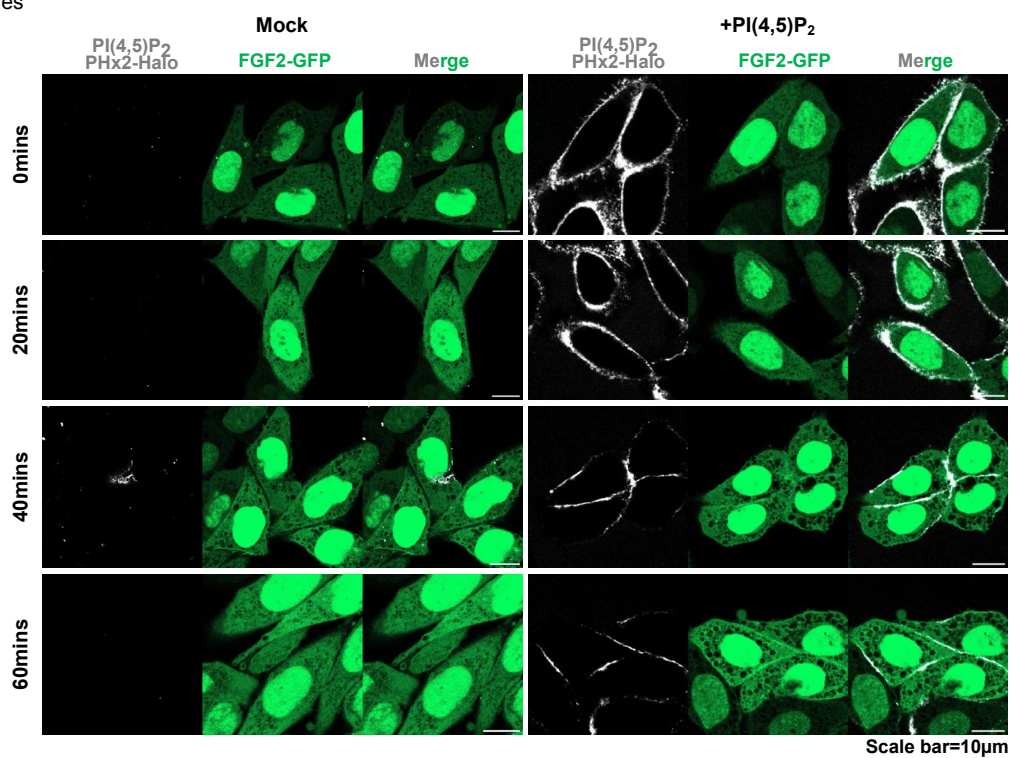

Uncropped images

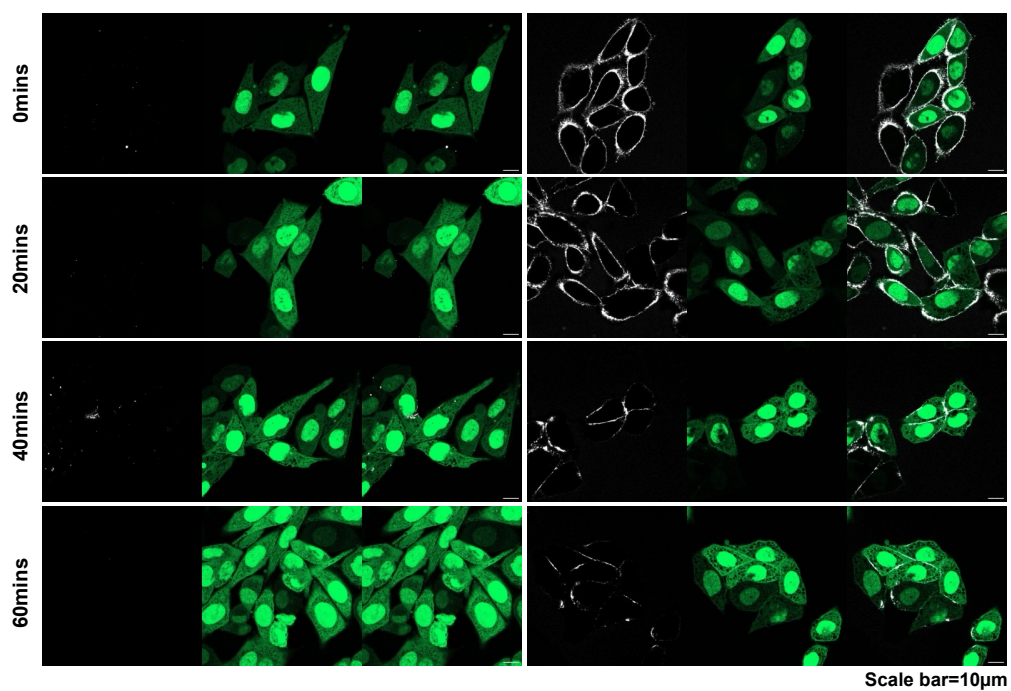

Supplement: Supplementary file 10 — SourceData [file 41467_2025_66860_MOESM10_ESM.zip › SourceData_NCOMMS-25-36735A/SorceData Figure 9 Cropped-Uncropped Confocal Images panel a.pdf]

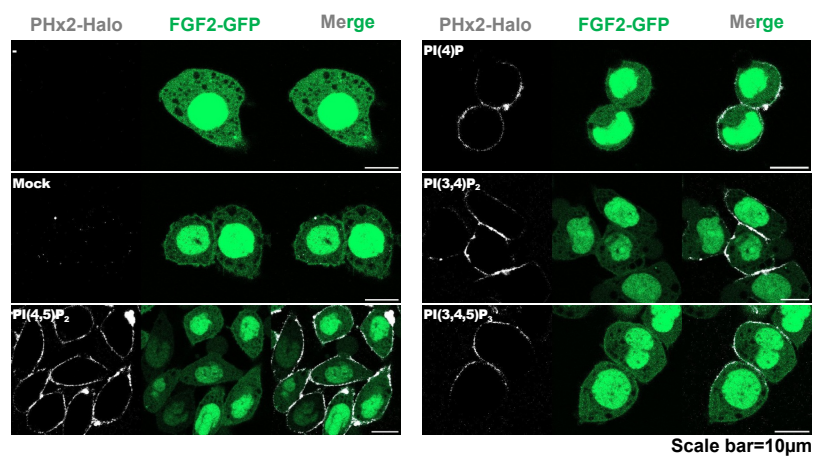

Supplement: Supplementary file 10 — SourceData [file 41467_2025_66860_MOESM10_ESM.zip › SourceData_NCOMMS-25-36735A/SourceData Figure 9 Confocal images.pdf]

Cropped images

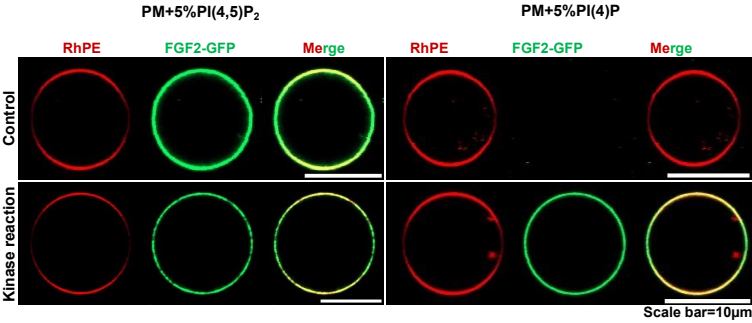

Uncropped images

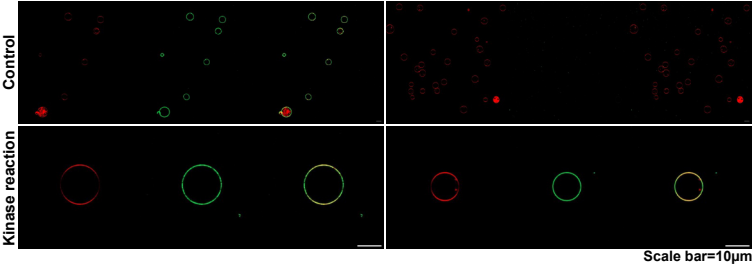

Supplement: Supplementary file 10 — SourceData [file 41467_2025_66860_MOESM10_ESM.zip › SourceData_NCOMMS-25-36735A/SorceData Figure 3 Cropped-Uncropped Confocal Images panel b and c.pdf]

Cropped images

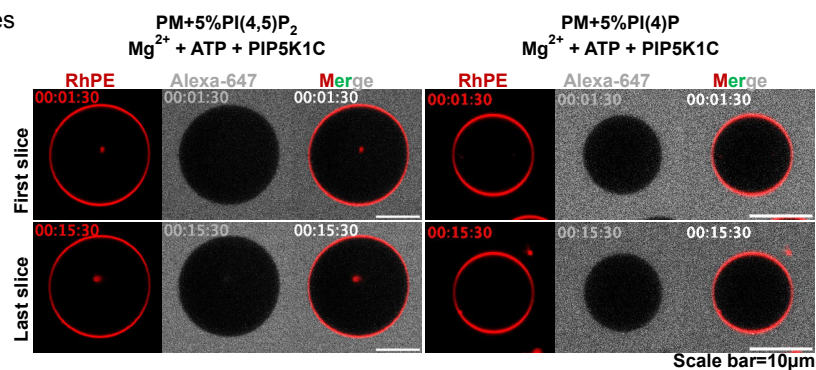

Uncropped images

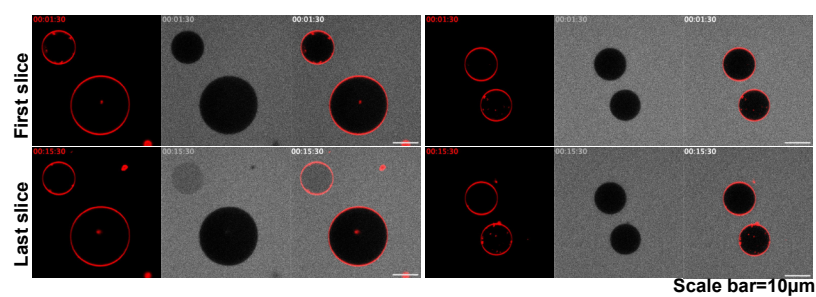

Supplement: Supplementary file 10 — SourceData [file 41467_2025_66860_MOESM10_ESM.zip › SourceData_NCOMMS-25-36735A/SorceData Figure 3 Cropped-Uncropped Confocal Images panel d and e.pdf]

**PM+5%PI(4,5)P<sub>2</sub>**

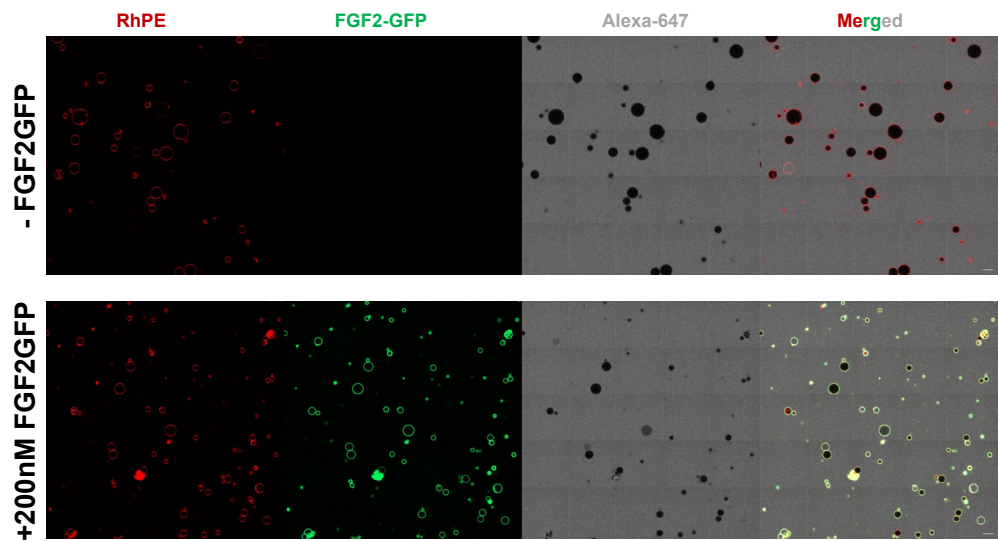

**PM+5%PI(4,5)P<sub>2</sub>\_Kinase reaction**

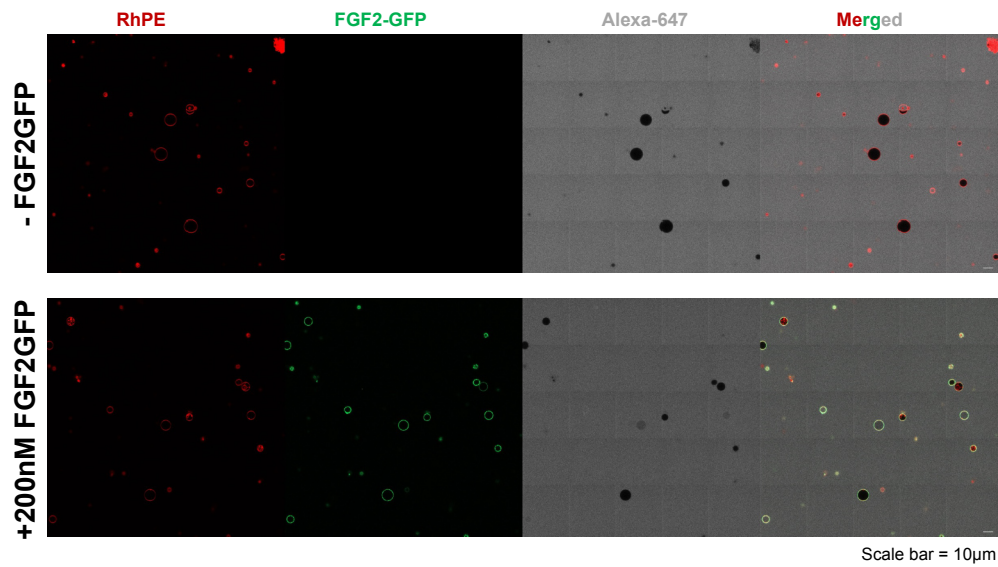

Supplement: Supplementary file 10 — SourceData [file 41467_2025_66860_MOESM10_ESM.zip › SourceData_NCOMMS-25-36735A/SourceData Figure 4 Confocal images panel d_2.pdf]

### PM+5%PI(4)P

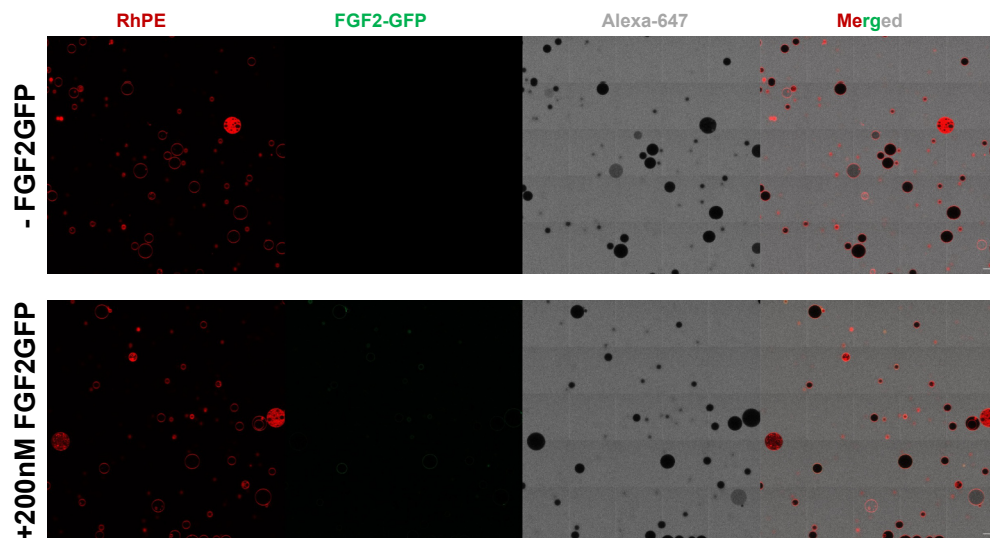

### PM+5%PI(4)P\_Kinase reaction without ATP

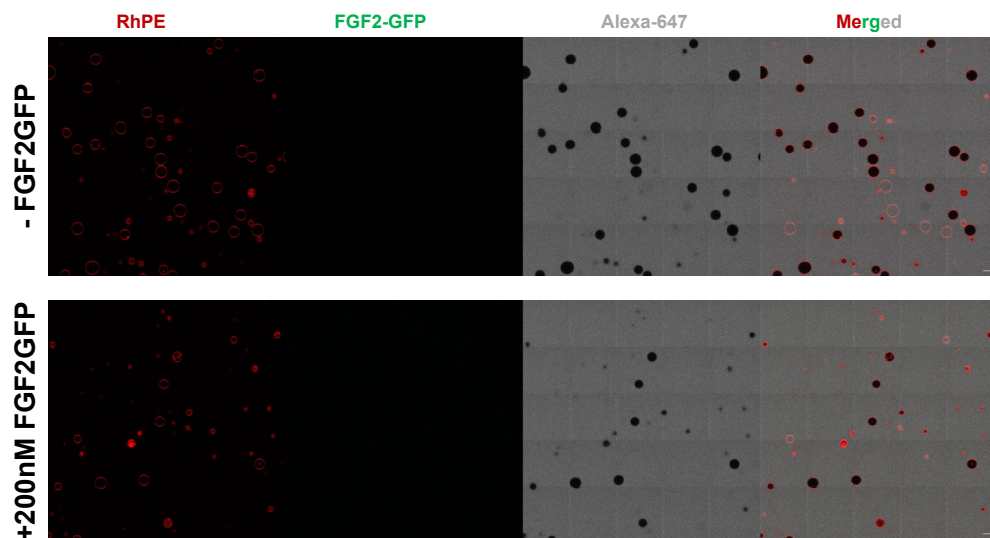

### PM+5%PI(4)P\_Kinase reaction

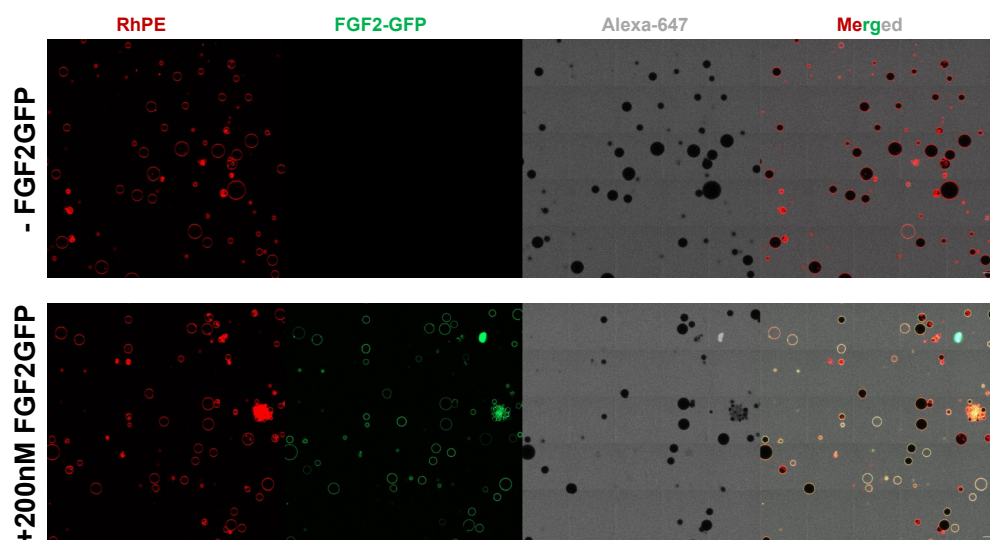

Supplement: Supplementary file 10 — SourceData [file 41467_2025_66860_MOESM10_ESM.zip › SourceData_NCOMMS-25-36735A/SourceData Figure 4 Confocal images panel d_1.pdf]

Cropped images

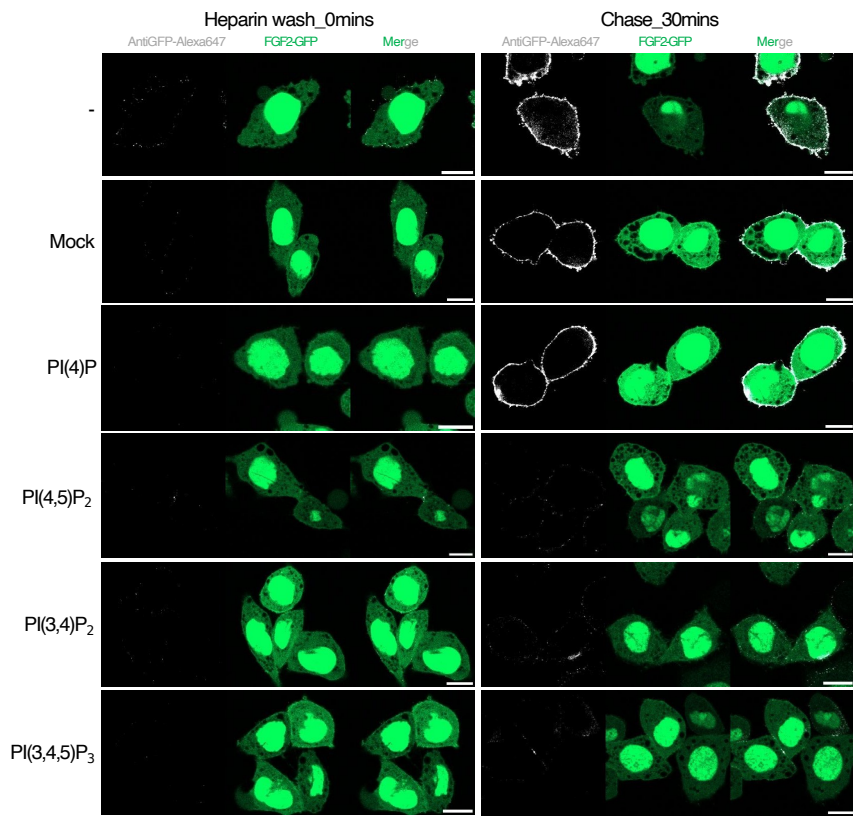

Uncropped images

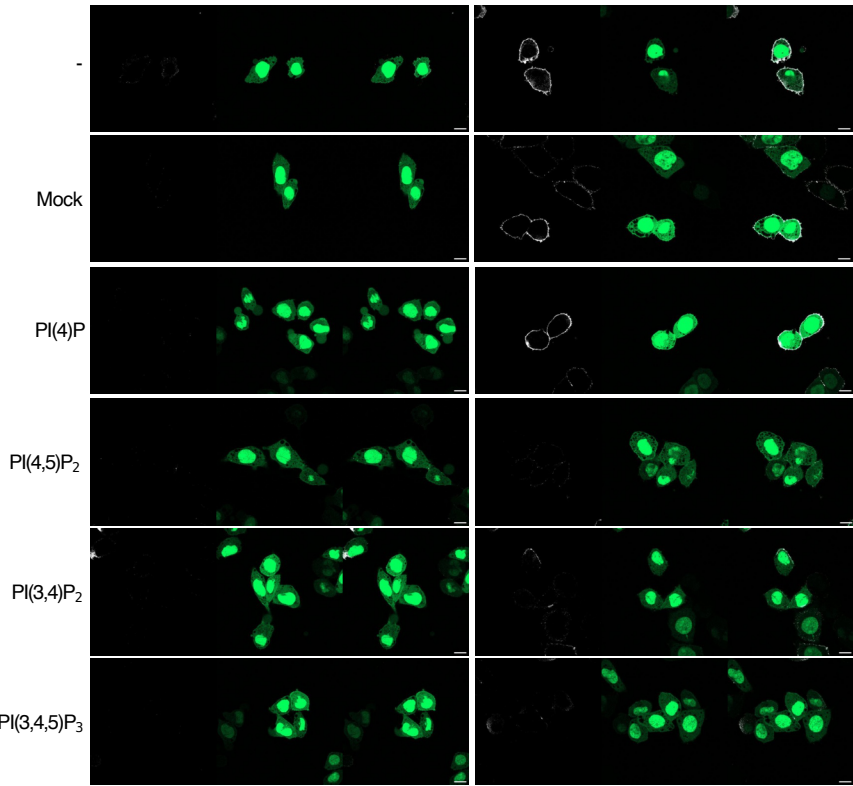

Supplement: Supplementary file 10 — SourceData [file 41467_2025_66860_MOESM10_ESM.zip › SourceData_NCOMMS-25-36735A/SorceData Figure 10 Cropped-Uncropped Confocal Images panel a.pdf]
